# Hypoglycemia as a cause of sudden cardiac arrest during cesarean delivery under spinal anesthesia: a case report and review of the literature

**DOI:** 10.1186/s13256-021-02976-2

**Published:** 2021-07-29

**Authors:** Temesgen Tilahun, Guteta Gudina

**Affiliations:** 1 Department of Obstetrics and Gynecology, Institute of Health Sciences, Wollega University, P.O Box: 395, Nekemte, Oromia Ethiopia; 2 Department of Anesthesia, Wollega University Referral Hospital, Nekemte, Ethiopia

**Keywords:** Cardiac arrest, Hypoglycemia, Spinal anesthesia, Case report

## Abstract

**Background:**

Sudden cardiac arrest during spinal anesthesia is a rare event. However, its management by an unprepared team is difficult and carries poor outcomes. Hypoglycemia as the cause of sudden cardiac arrest is rarely reported. This case illustrates lifesaving procedures for sudden cardiac arrest secondary to hypoglycemia during cesarean delivery under spinal anesthesia.

**Case summary:**

We report a case, from rural Ethiopia of sudden cardiac arrest secondary to hypoglycemia during cesarean delivery under spinal anesthesia. The case was successfully managed by a team of anesthetists and other operating teams. The mother and newborn were discharged from the hospital on the 7th postoperative day.

**Conclusion:**

Hypoglycemia during cesarean delivery under spinal anesthesia can cause sudden cardiac arrest. Therefore, identifying patients at risk of developing hypoglycemia, monitoring the patient’s condition, and initiating prompt intervention at the first sign of cardiovascular instability is advisable. Determining serum blood glucose levels at admission to the labor ward and monitoring blood glucose levels during spinal anesthesia should be routine practices.

## Introduction

Spinal anesthesia has become the preferred anesthetic technique when providing anesthesia for patients undergoing elective cesarean section as it carries less risk to the mother and fetus [1]. However, some severe complications can happen in spinal anesthesia [1]. The most common complications are cardiovascular in origin, hypotension being the most common [1, 2].

The other rare but serious complication of spinal anesthesia is sudden cardiac arrest [2]. In recent studies, the incidence of cardiac arrest during spinal anesthesia has been reported to be 2.5–6.4 per 10,000 anesthesia [1]. It is often difficult to determine whether surgical, anesthesia, or patient factors are the most significant factors leading to cardiac arrest [1, 2]. The possible factors that may cause cardiac arrest during spinal anesthesia in a case of cesarean section are related to the anesthetic procedure, perioperative significant blood loss, amniotic fluid embolism, metabolic disorders, and/or pathophysiological changes in the obstetric patient [1–4].

Here we present a case of successfully managed sudden cardiac arrest during cesarean delivery under spinal anesthesia secondary to hypoglycemia.

## Case presentation

This is a 25-year-old gravida 2 para 1(alive) from rural Ethiopia mother whose gestational age from reliable last normal menstrual period was 38 weeks. She was referred to Wollega University Referral Hospital with the diagnosis of prolonged labor. Her complaint at arrival to the hospital was labor pain of 15 hours and rupture of the membrane of 10 hours. She was fasting for the last 9 hours because of the pain. She received antenatal care twice at a nearby health center during which routine laboratory investigations such as blood group, urinalysis, complete blood count (CBC), serology for hepatitis B surface antigen, Venereal Disease Research Laboratory (VDRL) test for syphilis, and human immunodeficiency virus (HIV) test were done. Her blood group is 0+, urinalysis result was nonrevealing, CBC was normal, and serology for hepatitis B surface antigen, VDRL, and HIV was nonreactive. Obstetrics ultrasound examination showed normal intrauterine pregnancy (Table 1).

**Table 1:** Summary of laboratory investigations of the case of sudden cardiac arrest managed at Wollega University Referral Hospital, 2021

Time of investigation	Type of laboratory test	Result of the test
At admission	Blood group	0+
	CBC	White blood cell count = 9000 cells/μL; Red blood cell count = 4.1 million cells/μL Hematocrit = 36%; Platelet count = 153,000 cells/μL
	Urinalysis	Nonrevealing
	VDRL	Nonreactive
	HBsAg	Nonreactive
	ELISA for HIV	Nonreactive
	Obstetrics ultrasound	Gestational age of 39 weeks, estimated fetal weight= 3100 grams, and fundal placenta
Intraoperative	RBG^a^	60 mg
After operation	Hematocrit	33%
	Serum electrolytes (sodium, potassium, magnesium, chlorine, and calcium)	Sodium = 138 mmol/L Potassium = 4.1 mmol/L Magnesium = 0.96 mmol/L Chlorine = 99.7 mmol/L Calcium = 0.65 mmol/L
	Echocardiography index	Normal finding
	Electrocardiography index	Normal finding

^a^Done from blood sample taken at admission

*CBC* Complete blood count,* VDRL* Venereal Disease Research Laboratory,* HBsAg* Hepatitis B surface antigen,* ELISA* Enzyme-linked immunosorbent assay,* HIV* Human immunodeficiency virus,* RBG* Random blood glucose

She had one normal vaginal delivery at a health center 3 years back, and the child is alive. Her previous medical and surgical histories were unremarkable. There is no history of hypertension, diabetes mellitus, or renal or cardiac diseases in her family. She is a housewife. She has good social interaction and has no exposure to industrial waste products. She has no history of smoking cigarettes or drinking alcohol. She denied a history of ingestion of drugs before admission. The pregnancy was planned, wanted, and supported. Her weight and height were 67 kg and 160 cm, respectively.

On examination, she was in labor pain. She had dry buccal mucosa and sunken eyeballs. Her vital signs were blood pressure (BP) 100/70 mmHg, pulse rate (PR) 100 beats per minute, respiratory rate (RR) 18 breaths per minute, and temperature 36 °C. Lymph glandular system, chest, and cardiovascular system were normal. Abdominal examination showed term-sized uterus, longitudinal lie, cephalic presentation, fetal heartbeat 143 beats per minute, and three uterine contractions in 10 minutes each lasting 35–45 minutes. Pelvic examination revealed, 8-cm dilation of cervix, vertex presentation, grade 2 molding, right occiput-transverse position, station at zero, moderate Capute, and grade 1 meconium-stained amniotic fluid. There was no abnormality detected on integumentary and musculoskeletal systems. On neurologic examination, she was oriented to time, person, and place and had normal reflexes and no neurologic deficit. With the final diagnosis of cephalopelvic disproportion secondary to malposition, she was admitted to the labor ward. At admission, complete blood count and urinalysis were done and normal. The remaining blood sample was kept at the laboratory for possible further tests. However, random blood glucose was not determined because it was not routine practice at our hospital. She was prepared for emergency cesarean delivery and transferred to the operation room. She was preloaded with 1000 ml of normal saline.

In the operation room, spinal anesthesia was given between the third and fourth lumbar vertebral spaces. The anesthesia team used 12.5 mg hyperbaric bupivacaine. The patient was put in a supine position with the table tilt to the left. After preparing the surgical site, the baby was delivered within 6 minutes. The placenta was delivered by cord traction, and there was no significant bleeding. Unfortunately, the patient became restless. She became drowsy, not responding to oral commands, and there was associated sweating. Her blood pressure started to fall, and she became bradycardic. She also developed two episodes of seizures. There was no chest pain or cough. The anesthesia team tried to maintain oxygenation by face mask with Bain circuit. However, her condition deteriorated, and eventually, she developed apnea with sudden cardiac arrest. The operating team acted promptly to respond to this unexpected complication. We immediately intubated the patient and ventilated her with 100% oxygen. Cardiopulmonary resuscitation (CPR) was given. Fifty milliliters of 40% dextrose intravenous push was given. Luckily, our patient responded to 40% dextrose, and her vital signs and heart activity returned to normal. The surgery was completed successfully.

She delivered a male alive neonate weighing 3000 g with Apgar scores of 7 and 9 at 1st and 5th minute, respectively. At the end of the surgery, her vital signs were within the normal ranges and the chest was clear. Then, she was extubated and transferred to the recovery room. She recovered very smoothly with no residual features of cerebral hypoxia or anoxia.

From the collected blood sample before surgery, serum electrolytes (sodium, potassium, magnesium, chlorine, and calcium) were determined and found to be within normal ranges. However, blood glucose was reported to be 60 mg. This means the operation was started in a patient with underlying hypoglycemia. Further evaluation was conducted by the attending internist. Her electrocardiography (ECG) showed normal cardiac activity with no sign of ischemia, and echocardiography was normal. Both the mother and her newborn were discharged on the 7th postoperative day.

## Discussion

This is a case of sudden cardiac arrest during cesarean section under spinal anesthesia. Here we demonstrated possible differential diagnoses, and illustrated management of sudden cardiac arrest secondary to hypoglycemia, which is rarely reported as its cause.

Spinal anesthesia is considered to be a safe and preferred procedure for cesarean section, but, rarely, complications can occur in clinical practice [4]. One of the grave complications as demonstrated in this case is sudden cardiac arrest. The prevalence of cardiac arrest in pregnancy is 1 in 30,000 ongoing pregnancies [2]. Its prevalence during cesarean delivery is also rare [1, 2]. Despite its magnitude, when it happens, it is difficult to manage, particularly in a resource-limited setting and by a less experienced team [5].

The causes of sudden cardiac arrest during spinal anesthesia are related to anesthetic procedures, surgical procedures, hypotension, primary cardiac problems, massive pulmonary embolism, hypoglycemia, pathophysiological changes during pregnancy, and other related factors [1–10]. Here, we tried to consider these as the possible differential diagnosis.

One of the causes of cardiac arrest is related to anesthetic procedures. It could be high-level blocks, vagal reaction, anaphylactic shock, and regional anesthesia toxicity [1, 4, 5]. In this particular case, the anesthetic procedure was done correctly, the dose was calculated based on body weight, and the patient has no history of drug allergy. Therefore, the anesthetic procedure was not likely to cause cardiac arrest. However, giving spinal anesthesia in patients with underlying malnutrition, fasting, and dehydration can lead to cardiac arrest even in anesthetic procedures that are done appropriately [6]. Therefore, identifying these conditions in the patient before administration of spinal anesthesia is always crucial.

Hypotension is among the leading causes of cardiac arrest [3]. Pregnant patients are more susceptible to hypotension, with incidences ranging from 50% to 90% [1]. The major proposed mechanisms for hypotension during pregnancy are the exaggerated effects of spinal anesthesia in advanced pregnancy due to aortocaval compression caused by the gravid uterus, and the increased sensitivity of nerve fibers in pregnant patients to the effect of local anesthetics [7], probably due to chronic exposure of progesterone altering protein synthesis in nerve tissue.

During cesarean delivery, the major risk factors of hypotension are inadequate preloading, excessive intraoperative bleeding, inappropriate patient positioning, large-sized baby, maternal obesity, and anesthesia-related factors [1, 3, 5].

The other cause of sudden cardiac arrest is primary cardiac problems such as myocardial ischemia and arrhythmias [1, 2, 4, 5]. Though there were seizure attacks in our case, the patient was hemodynamically stable and well oxygenated before the administration of spinal anesthesia. She has also no known history of cardiac disease. No ischemic changes were noticed on ECG. Echocardiography was also normal. Therefore, in this case, primary cardiac problems as the cause of cardiac arrest are less likely.

Another possibility in our case is massive pulmonary embolism. When occurring after delivery, it accounts for cardiac arrest and has an unfavorable prognosis. Release of compression by the gravid uterus after delivery facilitates the movement of thrombi formed in blood vessels in the lower body to the pulmonary circulation, resulting in pulmonary embolism and decrease of blood flow to the left ventricle, inducing cardiac arrest [8, 9]. However, our patient had no respiratory complaints such as chest pain, cough, or hemoptysis, and the chest was clear, making the diagnosis less likely.

In this case, it seems that surgery-related metabolic changes played a significant role in causing sudden cardiac arrest. Every surgical procedure is associated with a stress response comprising endocrine and metabolic changes. Consequently, blood glucose concentrations will increase, even in the absence of preexisting diabetes [10]. On the contrary, glucose turnover in fasted pregnant women is several times greater than in nonpregnant women. Pregnant women are more prone to fasting hypoglycemia as there is continuous glucose utilization by the fetus that exaggerates the metabolic consequences of starvation. With brief maternal fasting before elective or emergency cesarean delivery, there is a more rapid fall in plasma glucose concentration [11–13]. Moreover, some spinal anesthetic agents have a glucose-lowering effect. A study conducted by Movaseghi among 150 parturients who took lidocaine spinal anesthesia showed a decrease in serum glucose levels compared with preoperative recordings [14]. All these factors could contribute to hypoglycemia in this particular case. There was also strong evidence to consider hypoglycemia as the cause of cardiac arrest. She was fasting for 9 hours. Intraoperatively, she had sweating and seizure attacks and her laboratory investigation showed hypoglycemia. She also responded to dextrose administration. All these factors could contribute to hypoglycemia in this particular case. There was also strong evidence to consider hypoglycemia as the cause of cardiac arrest. She was fasting for 9 hours. Intraoperatively, she had sweating and seizure attacks and her laboratory investigation showed hypoglycemia. She also responded to dextrose administration. Hypoglycemia can also be caused by infection, sepsis, and radiation [15–17]. However, in this case, there is no evidence to consider these as causes of hypoglycemia.

The mechanisms by which hypoglycemia causes sudden cardiac arrest and sudden death are incompletely understood. However, it is demonstrated in animal models that severe hypoglycemia-induced cardiac arrest and mortality can be caused by lethal cardiac arrhythmias or myocardial ischemia. Hypoglycemia can also cause seizures [18, 19]. The sympathetic nervous system has been hypothesized to be a major cause of fatal arrhythmias. Administration of nonselective β1- or β2-adrenergic blockade completely prevented severe hypoglycemia-induced cardiac arrhythmias and sudden death [18, 20]. Besides, treatment with anti-seizure medications reduces the incidence of sudden death during severe hypoglycemia [18].

Management of sudden cardiac arrest in a resource-limited setting is always difficult [6]. Therefore, preventive strategies should include appropriate patient selection for spinal anesthesia, maintaining adequate preload, prompt replacement of fluid and blood loss, and vigilance during patient positioning [6]. This case reminds the clinician to consider hypoglycemia in patients with sudden cardiac arrest and to attempt correcting low blood glucose if noted. However, random administration of glucose is not advisable as it carries poor neurologic outcomes [21].

## Conclusion

Hypoglycemia during cesarean delivery under spinal anesthesia can cause sudden cardiac arrest. Therefore, identifying patients at risk of developing hypoglycemia, monitoring the patient’s condition, and initiating prompt intervention at the first sign of cardiovascular instability is advisable. Determining serum blood glucose levels at admission to the labor ward and monitoring blood glucose levels during spinal anesthesia should be routine practices.

## Data Availability

The datasets used during the current study are available from the corresponding author on reasonable request.

## Abbreviations

CBC: Complete blood count
ECG: Echocardiography
ELISA: Enzyme-linked immunosorbent assay
HBsAg: Hepatitis B surface antigen
HIV: Human immunodeficiency virus
RBG: Random blood glucose
VDRL: Venereal Disease Research Laboratory
